# Efficient Direct Formic Acid Fuel Cells (DFAFCs) Anode Derived from Seafood waste: Migration Mechanism

**DOI:** 10.1038/s41598-017-17978-8

**Published:** 2017-12-19

**Authors:** Gumaa A. El-Nagar, Mohamed A. Hassan, Iver Lauermann, Christina Roth

**Affiliations:** 1 Chemistry Department, Faculty of Science, Cairo University, 12613 Cairo, Egypt; 2 Institute for Chemistry and Biochemistry, FU Berlin, Takustr. 3, D-14195 Berlin, Germany; 3 Nanotechnology and Advanced Materials Central Lab, Agriculture Research Center, Giza, Egypt; 4 Helmholtz-Zentrum Berlin für Materialien und Energie, Hahn-Meitner-Platz 1, 14109 Berlin, Germany

## Abstract

Commercial Pt/C anodes of direct formic acid fuel cells (DFAFCs) get rapidly poisoned by *in-situ* generated CO intermediates from formic acid non-faradaic dissociation. We succeeded in increasing the Pt nanoparticles (PtNPs) stability and activity for formic acid oxidation (DFAFCs anodic reaction) by embedding them inside a chitosan matrix obtained from seafood wastes. Atop the commercial Pt/C, formic acid (FA) is predominantly oxidized via the undesired poisoning dehydration pathway (14 times higher than the desired dehydrogenation route), wherein FA is non-faradaically dissociated to CO resulting in deactivation of the majority of the Pt active-surface sites. Surprisingly, PtNPs chemical insertion inside a chitosan matrix enhanced their efficiency for FA oxidation significantly, as demonstrated by their 27 times higher stability along with ~400 mV negative shift of the FA oxidation onset potential together with 270 times higher CO poisoning-tolerance compared to that of the commercial Pt/C. These substantial performance enhancements are believed to originate from the interaction of chitosan functionalities (e.g., NH_2_ and OH) with both PtNPs and FA molecules improving FA adsorption and preventing the PtNPs aggregation, besides providing the required oxygen helping with the oxidative removal of the adsorbed poisoning CO-like species at low potentials. Additionally, chitosan induced the retrieval of the Pt surface-active sites by capturing the *in-situ* formed poisoning CO intermediates via a so-called “migration mechanism”.

## Introduction

Direct formic acid fuel cells (DFAFCs) are an auspicious and robust alternative power concept for portable applications (*e.g*., laptops, cameras, phones, etc), thanks to their unique features including higher energy density and open circuit potential, lower toxicity and fuel crossover, better oxidation kinetics, greater energy conversion efficiency and better safety compared to the traditional hydrogen and methanol fuel cells^1–8^. Nevertheless, they still suffer from their insufficient long-term stability that obviously restricts their overall commercialization prospect. Unfortunately, this results from the fast deactivation of their commercial Pt-based catalysts with *in-situ* generated carbon monoxide (CO) intermediates from the “*non-faradaic*” dehydration of formic acid at the Pt surface^1,3,9–11^. Thus, the development of an efficient and stable DFAFCs anode catalyst for formic acid electrooxidation (FAO) with a high CO poisoning tolerance is a key requirement for their commercialization and practical application.

Extensive research efforts have been devoted to mitigate such poisoning effects (i.e., CO poisoning) to improve the Pt-based catalysts performance through several strategies. One of these approaches, is alloying platinum nanoparticles (PtNPs) with non-precious metals/metal oxides to facilitate the oxidative removal of adsorbed CO at low potentials via providing required oxygen-containing functionalities (i.e., bifunctional mechanism). The ensemble effect is another strategy to mitigate CO poisoning via resisting the CO adsorption through distorting the structural proximity of the Pt surface active-sites required for CO adsorption/formation^12–16^. Another possible pathway is to modify the Pt surface electronic structure in such a way that it disfavors the CO adsorption through a so-called “electronic effect”^1,9,17–22^.

Another severe drawback of DFAFCs is the degradation of their commercial Pt- and Pd-based electrocatalysts resulting from the agglomeration (through diffusion and/or coalescence), sintering (via dissolution/re-deposition process) and corrosion of the support material (N.b., Pd and Pt nanoparticles facilitate the oxidation of the carbon support material), resulting in a permanent loss of DFAFCs performance^23–25^. Thus, the carbon support materials play a central role in improving the utilization and catalytic activity of the precious metal nanocatalysts. In this context, another efficient strategy to reduce the cost and to enhance the performance of the Pt-based catalysts, is anchoring the PtNPs on supporting materials that could not only provide large surface area, but also improve their performance (both activity and stability) through catalyst-support interactions. Several advanced carbon materials with high specific surface area, excellent physicochemical stability and electrical conductivity, such as carbon nanotubes and graphene, are commonly used to anchor the Pt nanoparticles^26–29^. However, despite these valuable efforts, the performance of the commercially-applied catalysts in DFAFCs remains insufficient and further improvement is required.

Herein, PtNPs embedded in a nano-chitosan matrix are introduced as alternative efficient and stable anode catalyst for DFAFCs. Chitosan is a natural polysaccharide biopolymer obtained from the deacetylation of chitin, which is the second most abundant natural polysaccharide and the major component of crustacean shells (e.g., shrimps, crabs, etc)^3,30^. We found that chitosan effectively reduces the average PtNPs size, improves the PtNPs dispersion, preventing the PtNPs sintering under the harsh electrochemical conditions through the strong interaction between the chitosan functionalities and PtNPs. It also facilitates the FAO charge transfer. Chitosan –OH and –NH_2_ like functionalities are believed to provide anchoring sites for the Pt nanoparticles facilitating their dispersion and avoiding their aggregation (i.e., chitosan adsorbed on the PtNPs surface results in the formation of a shell that stabilizes and protects the PtNPs). Besides, chitosan functional groups are believed to re-activate the poisoned PtNPs surface sites with the strongly adsorbed CO intermediates by capturing these adsorbed CO species from the PtNPs surface via a so-called “migration phenomenon”. Additionally, chitosan is believed to modify the PtNPs electronic structure in such a way that it disfavors the poisoning dehydration pathway and provides the required oxygen-containing groups needed for the oxidative removal of CO at low potentials. To the best of our knowledge, this is the first report on the electrochemical properties of the as-prepared nano-composite for DFAFCs applications.

## Experimental

### Chemicals

Chloroplatinic acid hydrate (>99.99%), sodium borohydride (~98%), formic acid (95–97%), acetic acid glacial (>98.85%), chitosan (molecular weight 110,000–150,000, purity ≥93%), and sulfuric acid were all purchased from Sigma-Aldrich and used without further purification. All solutions were prepared using deionized water with resistivity of 18.2 MΩ cm which was prepared using a Milli-Q reagent deionizer (Millipore).

### Catalysts and Electrodes Preparation

Chitosan-PtNPs nanocomposite (nano-chitosan-PtNPs) was prepared using a simple chemical reduction method as follows: firstly, chitosan solution (2 mg/ml) was prepared in 1% acetic acid aqueous solution, as described in our previous publications^30^. Then, 3 ml of the above prepared chitosan solution was mixed with 50 μl of chloroplatinic acid aqueous solution (0.5 M) and the mixture was stirred for at least 5 hours. Next, 250 μl of a freshly prepared sodium borohydride aqueous solution (1 M) was added to the above mixture and stirred for 4 hours until the complete reduction of the platinum salt (N.b., sodium borohydride concentration was selected ~10 times higher than that of the metal salt to obtain the complete reduction of all metal salts). Various volumes of platinum salt were used to prepare chitosan-PtNPs composites with different Pt weight percentages. Finally, 10 μl of the above-prepared chitosan-PtNPs nanocomposite was cast onto the mechanically polished glassy carbon electrode (GCE) surface and left to dry at 50 °C for about 30 min to get a deposited catalyst layer with 19 μg/cm^2^ loading. The same procedures were used to prepare the nano-chitosan without adding any Pt salt to the chitosan solution. Pt nanoparticles (PtNPs) were prepared also using the above-mentioned chemical reduction method by adding NaBH_4_ solution to 0.5 M chloroplatinic acid aqueous solution (without chitosan).

The commercial Pt/C (20 wt%) modified GCE was prepared by drop-casting 5 μl of Pt/C suspension (0.024 g suspended in 2 ml isopropanol/water (1:1) and one-drop Nafion solution (25%)) onto the GCE surface resulting in 85 μg/cm^2^ for comparison.

### Electrochemical and Materials Characterizations

All electrochemical experiments were performed in a three-electrode conventional glass cell using a Gamry Potentiostat/Galvanostat Reference 3000 setup with electrochemical impedance spectroscopy (EIS) unit. Glassy carbon electrode (GCE, diameter ~3 mm), SCE and spiral Pt electrodes served as working, reference and counter electrodes, respectively. The electrocatalytic activity of the as-prepared catalysts was investigated in 0.5 M H_2_SO_4_ containing 0.3 M formic acid. High resolution transmission electron microscope (HR-TEM, Tecnai G20, FEI, Netherland) and scanning electron microscope coupled with an energy dispersive X-ray spectrometer (SEM/EDS, HITACHI UHR FE-SEM SU8030) were used to evaluate the electrode morphology and composition. X-ray diffraction in transmission geometry (STOE STADI-P) operated with Cu K_α_ radiation (λ = 1.54 Å) and position sensitive detector was used to identify the change in the particle size and the crystallographic structure of the as-prepared catalysts. X-ray photoelectron spectroscopy (XPS, CLAM4 electron analyzer from Thermo VG scientific), using a Mg Kα X-ray source (1254 eV) was used to determine the samples chemical (surface) composition. For evaluation, a linear background was subtracted and peaks were fitted using Voigt functions with identical FWHM for each component of the same element.

### DFT calculations

The interaction of FA, CO and PtNPs with the chitosan matrix is modeled by a density functional theory (DFT) approach using Gaussian 09 suite of programs. All the structures were optimized at Density Functional three-parameter hybrid (B3LYP) level theory by implementing the 6–311++G(d, p) basis set for all the atoms except Pt atom, for which the LanL2DZ basis set was used^2^.

## Results and Discussions

### Materials characterization

**Figure 1:**
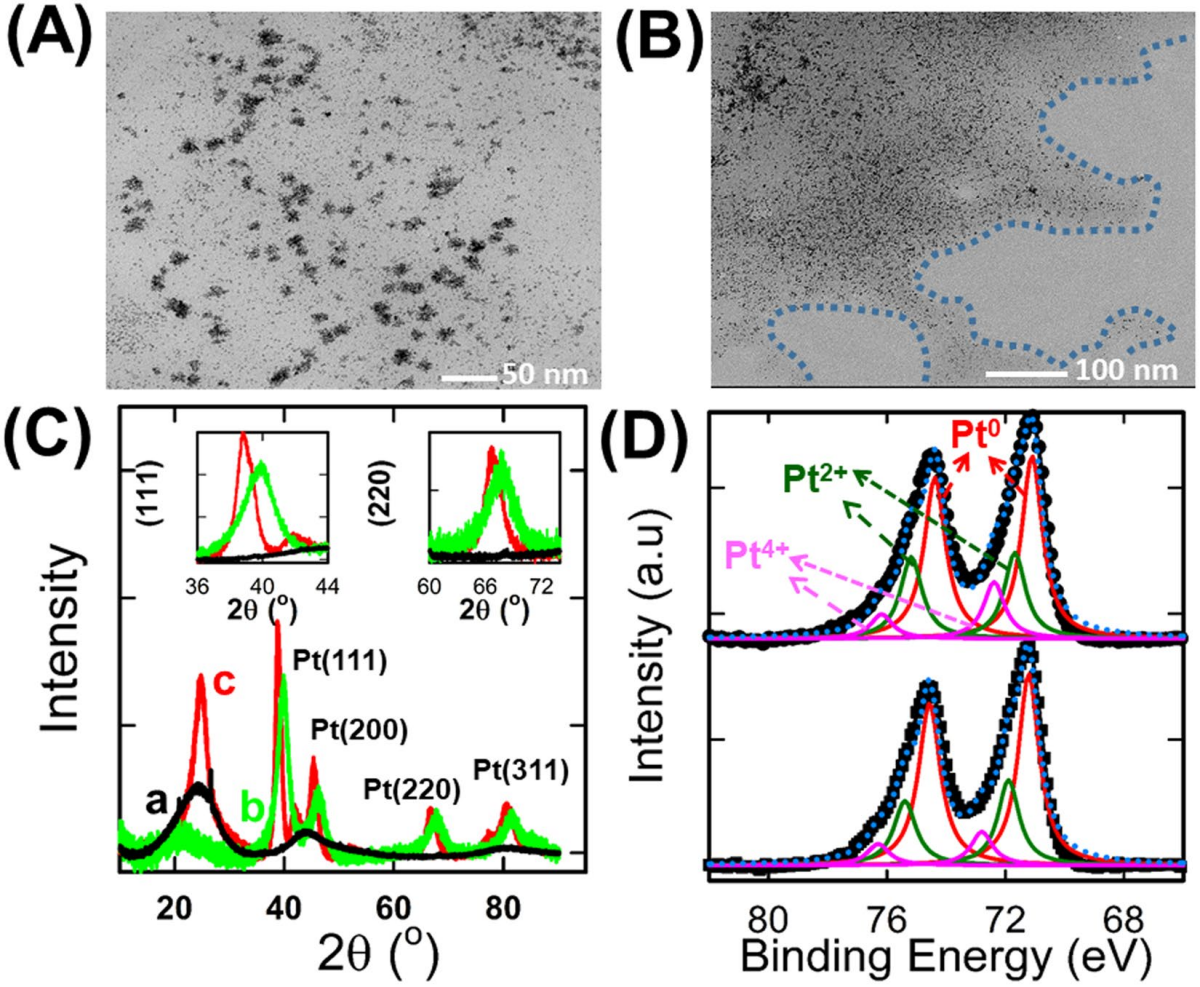
**(A** and **B**) TEM images of PtNPs (**A**) and nano-chitosan-PtNPs (**B**). (**C**) XRD patterns of nano-chitosan (a), nano-chitosan-PtNPs (b) and PtNPs (c) catalysts. (**D**) Pt4f high resolution XPS spectra of PtNPs (upper-panel) and nano-chitosan-PtNPs (lower -panel).

The morphology, average particle size and the composition of the as-prepared catalysts were first examined by TEM, SEM and EDS techniques. As seen in Fig. 1(A and B), the chemical reduction of PtNPs in the absence of nano-chitosan matrix (assigned as PtNPs) resulted in inhomogeneously distributed and aggregated Pt particles with an average particle size of ca. 20 nm (as shown in Image A). But their insertion inside the nano-chitosan matrix (denoted as nano-chitosan-PtNPs) exhibited a homogeneous distribution of spherical PtNPs having an average particle size of ~3 nm in the chitosan matrix (i.e., nano-chitosan formed a thin coating layer on individual Pt nanoparticles, Image B). Moreover, nano-chitosan-PtNPs have a sponge-like structure with well-distributed PtNPs over the entire nano-chitosan sponge-like matrix as revealed from its SEM and mapping EDS analysis, as shown in Fig. S1. Additionally, its EDS spectrum displayed the characteristic peaks of the nano-chitosan building blocks (carbon, nitrogen and oxygen peaks), besides PtNPs peaks (with 5% PtNPs weight percent, See Fig. S1). This indicates the successful insertion of the PtNPs into the chitosan matrix via a simple chemical reduction method. It also shows the essential role of the chitosan matrix in the distribution and stabilization of the PtNPs (i.e., it prevents the PtNPs aggregation). Nano-chitosan alone showed an irregularly-shaped structure and its corresponding EDS analysis exhibited only carbon, oxygen and nitrogen peaks which are attributed to the chitosan structure units (See Fig. S2).

The crystallographic orientation of the Pt nanoparticles  in the presence (nano-chitosan-PtNPs) and absence of nano-chitosan (PtNPs) was further examined via XRD, data are presented in Fig. 1C. Both PtNPs (curve c) and nano-chitosan-PtNPs (curve b) electrodes unambiguously exhibited the typical face-centered cubic (fcc) Pt structure (JCPDS-NO. 04-0802), where the diffraction peaks at 38.57°, 45.15°, 66.35°, 80° and 84.7° can be indexed to Pt (111), Pt (200), Pt (220), Pt (311) and Pt (222) reflections, respectively. The diffraction peaks located at 24.8°, 41.9°, 51.7° and 77.1° for both the nano-chitosan (curve a) and nano-chitosan-PtNPs can be indexed to the C (002), C (101), C (004) and C (110) planes of the carbon support, respectively. Interestingly, the PtNPs diffraction peaks of nano-chitosan-PtNPs are slightly shifted positive, suggesting changing of the PtNPs lattice distances attributed to the chitosan shell stress or strain on the PtNPs crystal lattice, as revealed in Fig. S3.

XPS measurements were carried out to analyze the surface composition and the valence states together with assessing the interfacial interaction between the nano-chitosan matrix and PtNPs, results are presented in Fig. 1D. Both the PtNPs (Fig. 1D- upper-panel) and nano-chitosan-PtNPs (Fig. 1D- down-panel) displayed the typical Pt 4f doublet peak around 71.3 and 74.6 eV^31,32^.

Each Pt4f peak can be deconvoluted into three doublets. The stronger peaks around 71.1 eV and 74.4 eV can be assigned to Pt° (4f_7/2_) and Pt° (4f_5/2_), respectively, and the peaks located at 72.4 and 76.5 can be indexed to Pt^2+^ (4f_7/2_) and Pt^2+^ (4f_5/2_), respectively. Whereas the weaker peaks at 75.6 eV and 78 eV can be attributed to Pt^4+^ (4f_7/2_) and Pt^4+^ (4f_5/2_), respectively. This indicates the presence of PtO or Pt(OH)_2_ in the surface layer according to the literature^32^. The Pt° is the predominant species in both PtNPs and nano-chitosan-PtNPs catalysts based on their relative integrated peak intensity.

Interestingly, the 4f_5/2_ and 4f_7/2_ peaks of chitosan-PtNPs nanocomposite are shifted to higher binding energy (71.3 eV and 74.8 eV) compared to that of 4f_5/2_ and 4f_7/2_ peaks of PtNPs (71.08 eV and 74.4 eV). This observed binding energy upshift suggests strong electronic interactions between the PtNPs and the chitosan structure resulting in a downshift of the Pt atoms d-band center position. This would positively affect the NPs electrocatalytic activity and decrease the CO binding strength on the PtNPs surface. This is in good agreement with the negative shift observed for the CO stripping peak in electrochemical measurements obtained at chitosan-PtNPs nanocomposite compared to that of PtNPs. The high resolution XPS C1s spectrum of the nano-chitosan-PtNPs can be deconvoluted into four well defined peaks at 285 eV (assigned to C-C, C-H and C-NH2), 287 eV (indexed to C-O and C=N), 288.3 eV (attributed to C=O and N-C-O) and 289 eV (assigned to O=C-O and O-C-O)^3^, see Fig. S4A. Furthermore, the high resolution XPS N1s spectrum of nano-chitosan-PtNPs shows a major peak at 400.5 eV which originates from the non-protonated amine or amide (–NH_2_ and –NH–) and a minor peak located at 401.7 eV, which is attributed to the protonated amine (–NH_3_^+^), see Fig. S4B.

### Electrocatalytic Activity for Formic Acid Oxidation (FAO)

**Figure 2:**
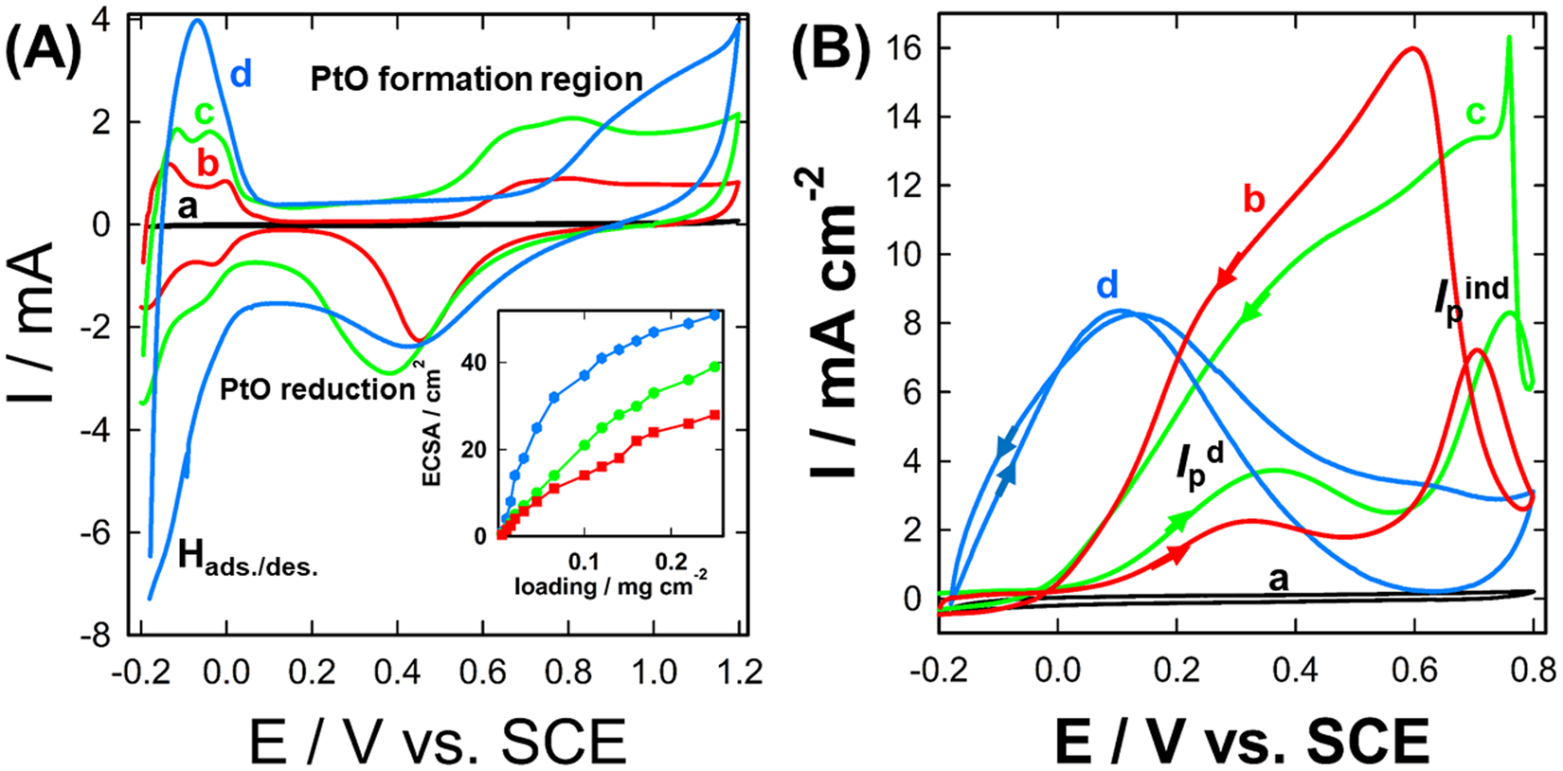
CVs measured at nano-chitosan (curve a, black-line), PtNPs (curve b, red-line), Pt/C (curve c, green-line) and nano-chitosan-PtNPs (curve d, blue-line) modified GCE electrodes in 0.5 M H_2_SO_4_ in the absence (**A**) and presence of 0.3 M FA (**B**- same color code as in **A**) with a potential scan rate of 50 mV/s. Inset of **A** shows the variation of electrochemical active surface area of PtNPs, Pt/C and nano-chitosan-PtNPs electrodes with their respective loadings (same color code as in **A**).

The electrochemical characterization of the as-prepared catalysts was performed first in aqueous 0.5 M H_2_SO_4_ solution to evaluate the PtNPs purity, facet’s densities and electrochemically active surface area (ECSA). As seen in Fig. 2A, the chitosan modified GCE (assigned as nano-chitosan/GCE) is featureless under the applied conditions (curve a). However, the PtNPs (curve b), commercial Pt/C (curve c) and nano-chitosan-PtNPs (curve d) modified GCE electrodes exhibited the typical voltammetric features of clean polycrystalline Pt in acid medium. All electrodes displayed the characteristic hydrogen adsorption/desorption region (H_ads/des_) in the potential range −0.2-0.0 V and Pt oxide (PtO) formation starting at potentials >0.6 V, which is coupled with a PtO reduction peak around ~0.4 V. while both PtNPs/GCE (curve b) and Pt/C (curve c) exhibited two well-defined peaks located at ~−0.12 V and ~−0.04 V attributed to hydrogen adsorption/desorption at Pt (110) and Pt (100) facet sites, respectively^1,17,21^.

Nano-chitosan-PtNPs modified GCE showed only one intense peak located at ~−0.08 V assigned to the Pt(111) facets. This suggests a polycrystalline structure of both PtNPs/GCE and Pt/C in contrast to a (111) facet-rich structure of the nano-chitosan-PtNPs. Furthermore, the PtNPs electrochemically active surface area (ECSA) of all the studied electrodes with various loadings was estimated from the hydrogen desorption charge using the standard reported value of 210 μC cm^−2^, the results are presented in the inset of Fig. 2A.

As revealed in this figure, the nano-chitosan-PtNPs nanocomposite modified GCE (assigned as nano-chitosan-PtNPs/GCE) exhibited a higher ECSA compared to that of the commercial Pt/C and PtNPs modified GCE electrodes over all the studied loading ranges. For instance, nano-chitosan-PtNPs/GCE exhibits 2 and 3 times higher ECSA compared to the Pt/C and PtNPs modified GCE electrodes at a Pt loading of 0.08 mg cm^−2^, respectively. This indicates the crucial role of the chitosan matrix in the formation of smaller well-dispersed PtNPs with higher number of Pt active surface-sites.

The electrocatalytic activity of the as-prepared electrodes for formic acid oxidation (FAO) was further investigated by recording their respective CVs in 0.5 M H_2_SO_4_ aqueous solution containing 0.3 M formic acid, as shown in Fig. 2B. Both PtNPs (curve b) and commercial Pt/C (curve c) modified GCE electrodes exhibited the typical behavior of FAO dual-mechanism at a clean polycrystalline Pt surface, with the appearance of two oxidation peaks at ~0.3 V and ~0.72 V in the positive-going potential scan^1,3,10,11,17,21,33–35^. Those two peaks are ascribed, respectively, to the direct pathway (in which FA is directly dehydrogenated to CO_2_, assigned as I_p_^d^) and indirect/poisoning route (in which the generated adsorbed CO from the *non-faradaic dissociation* of FA are being oxidized to CO_2_, denoted as I_p_^ind^). As clearly seen in Fig. 2A (curves b & c), FA is oxidized predominantly through the undesired indirect route (dehydration/poisoning pathway) at both PtNPs (curve b) and Pt/C (curve c) modified GCE electrodes as indicated from the relative current densities associated with the I_p_^d^ and I_p_^ind^ peaks. That is, the I_p_^ind^ has a much higher intensity compared to that of I_p_^d^, indicating the extensive poisoning of most of the PtNPs surface-active sites with the CO which is *in-situ* produced from the dehydration of FA. It is worth to be mentioned here that the oxidation current of FA is significantly improved in the reverse scan direction (assigned as I_b_) attributed to the FA oxidation at the poison-free Pt surface sites obtained from the oxidation of CO at I_p_^ind^ peak together with the availability of the Pt-Ox, which enhanced the current in this region. Interestingly, the PtNPs chemically embedded inside the chitosan matrix (nano-chitosan-PtNPs) enhanced the FA oxidation performance as evaluated from the exclusive oxidation of FA via the direct pathway (indirect peak completely disappeared) together with a large negative shift of the FA oxidation onset potential (~400 mV), see Fig. 2A (curve d), relative to both commercial Pt/C and PtNPs modified GC electrode with the same loading. Furthermore, two parameters (namely, I_p_^d^/I_p_^ind^ and I_p_^d^/I_b_) are used to probe the performance of the developed catalysts for FA oxidation and their CO poisoning tolerance.

The I_p_^d^/I_p_^ind^ of the chitosan-PtNPs is ca. 270 times higher compared to that of the PtNPs and Pt/C modified GCE, reflecting the superiority of the nano-chitosan-PtNPs/GCE catalyst for the direct formic oxidation to CO_2_. In addition, the I_p_^d^/I_b_ of the chitosan-PtNPs catalyst is 10 and 5 times higher compared to the PtNPs and Pt/C modified GCE, indicating the higher CO-poisoning tolerance of nano-chitosan-PtNPs/GCE catalyst. Our proposed electrocatalyst (chitosan-PtNPs) shows a higher or comparable electrocatalytic activity and durability compared to commercial Pt-based electrodes, as well as the most latest reported electrocatalysts for formic acid electrode oxidation (see Table S1-supplementary information).

**Figure 3:**
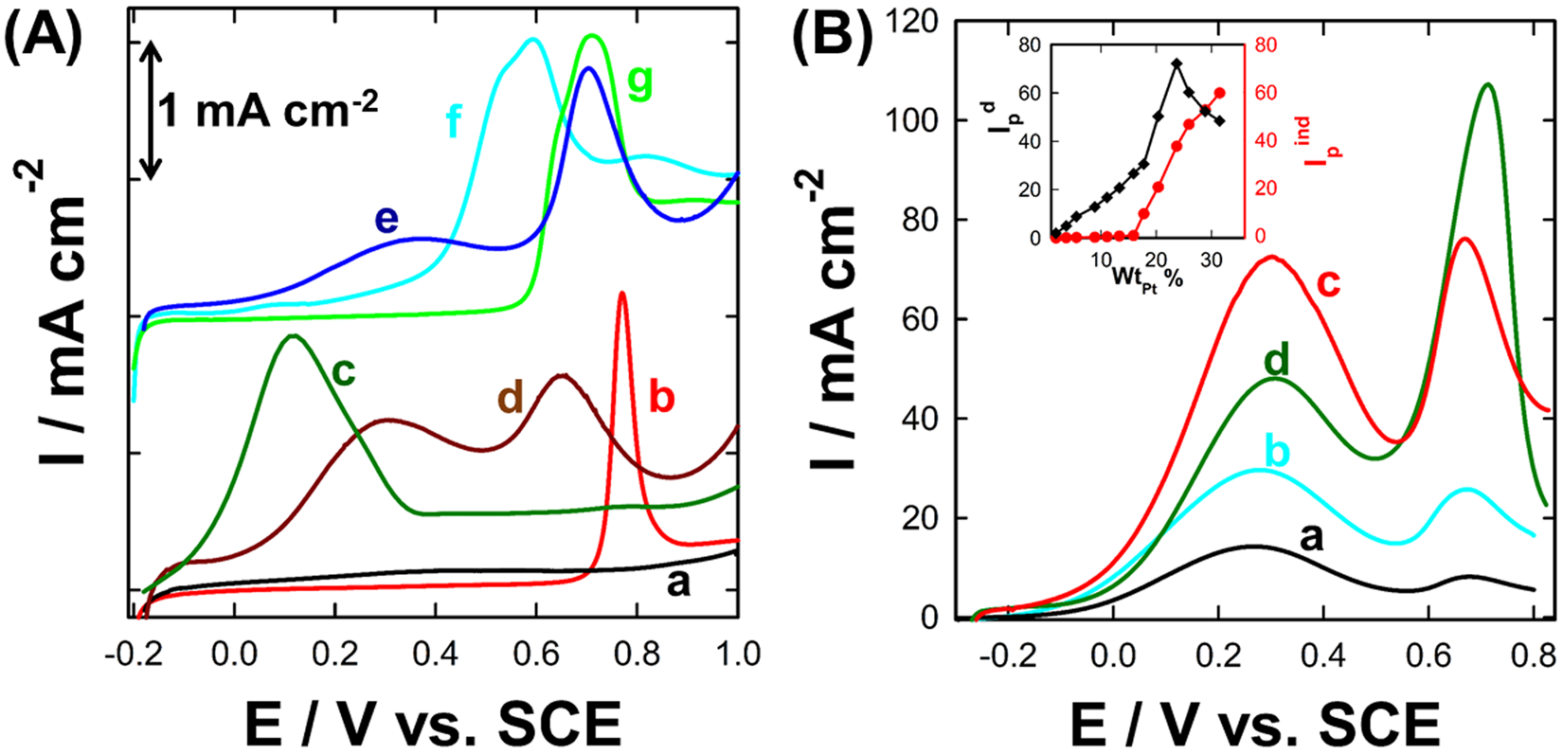
(**A**) CO stripping at (a) nano-chitosan, (b) PtNPs, (c–g) nano-chitosan-PtNPs with various PtNPs compositions (typically, (c) 10%, (d) 18%, (e) 25%, (f) 30% and (g) 35%) modified GCE in 0.5 M H_2_SO_4_ with a scan rate of 50 mV/s. (**B**) LSVs measured at nano-chitosan-PtNPs/GCE with different PtNPs weight percent (namely, (a) 15%, (b) 20%, (c) 30% and (d) 35%) in 0.5 M H2SO4 with a potential scan rate of 50 mV/s.

This evokes several questions: What is the role of the chitosan matrix in the observed enhancement? Did the chitosan matrix resist the CO adsorption and/or formation via a *third-body effect*? Or did it catalyze the CO oxidative removal at low potentials through the *bifunctional and/or electronic effec*t? To answer these questions, a CO monolayer was allowed to chemisorb *non-faradaically* at open circuit potential atop of the as-prepared catalysts. Then, the adsorbed CO layer was voltammetrically stripped off in 0.5 M H_2_SO_4_ aqueous solution, see Fig. 3A. This figure reveals the following interesting details:

(I) No CO stripping peak is observed at the nano-chitosan/GCE (curve a), while a strong CO oxidative removal peak appeared at 0.77 V for the PtNPs/GCE (curve b), indicating that chitosan cannot initiate the oxidation of FA and its *non-faradaic* dissociation to CO, while the PtNPs sites are essential for FA adsorption and oxidation.
(II) Interestingly, the nano-chitosan-PtNPs/GCE (10% Pt, curve c) exhibited one broad CO oxidation peak with a strong negative shift of its onset potential (~600 mV) compared to that of PtNPs/GCE (curve b). This incredible negative shift in both the FA and CO oxidation onset potentials will result in an auspicious performance improvement (especially stability see forward the details in section 3.3) of DFAFCs and supports consequently a significant modification of the PtNPs surface electronic structure upon embedding them inside the chitosan structure. This strategy most possibly weakens the Pt-CO binding so that CO oxidation commences at low potentials which is in good agreement with the XPS (Fig. 1D) and XRD results (Fig. 1C). Alternatively, chitosan’s OH-functional groups might be providing the required oxygen-containing groups to facilitate the CO oxidative removal at earlier polarization potentials by the so-called *bifunctional effect*.
(III) Surprisingly, the amount of the stripped CO from nano-chitosan-PtNPs/GCE (10% Pt, curve c) was ca. 3 times higher than that of the PtNPs/GCE (with the same PtNPs loading and area) as calculated using the amount of charge associated with CO oxidation. Based on this observation, the *third-body* rationalization can be safely excluded from the catalytic improvement. Actually, if it works, the amount of stripped adsorbed CO from the chitosan-PtNPs catalyst would certainly get lowered, which is not the case here. The reason for the observed behavior is still unclear. Why does the amount of stripped CO at the nano-chitosan-PtNPs increase relative to that of the PtNPs with the same area and loading?
(IV) Unpredictably, two adsorbed CO oxidation peaks at 0.25 V and 0.65 V (curve d) were observed, when the amount of the embedded PtNPs inside the nano-chitosan matrix increases up to 18%. In addition, a further increase of the embedded PtNPs (e.g. 25% and 30%, see curves e-f) resulted in a significant increase of the second peak at the expense of the first peak (at 0.25 V). Furthermore, the further increase of PtNPs loading beyond 35% resulted in the appearance of only one oxidation peak around 0.67 V (curves g) which is still negatively shifted (~100 mV) compared to that of the pure PtNPs (curve b).

**Figure 4:**
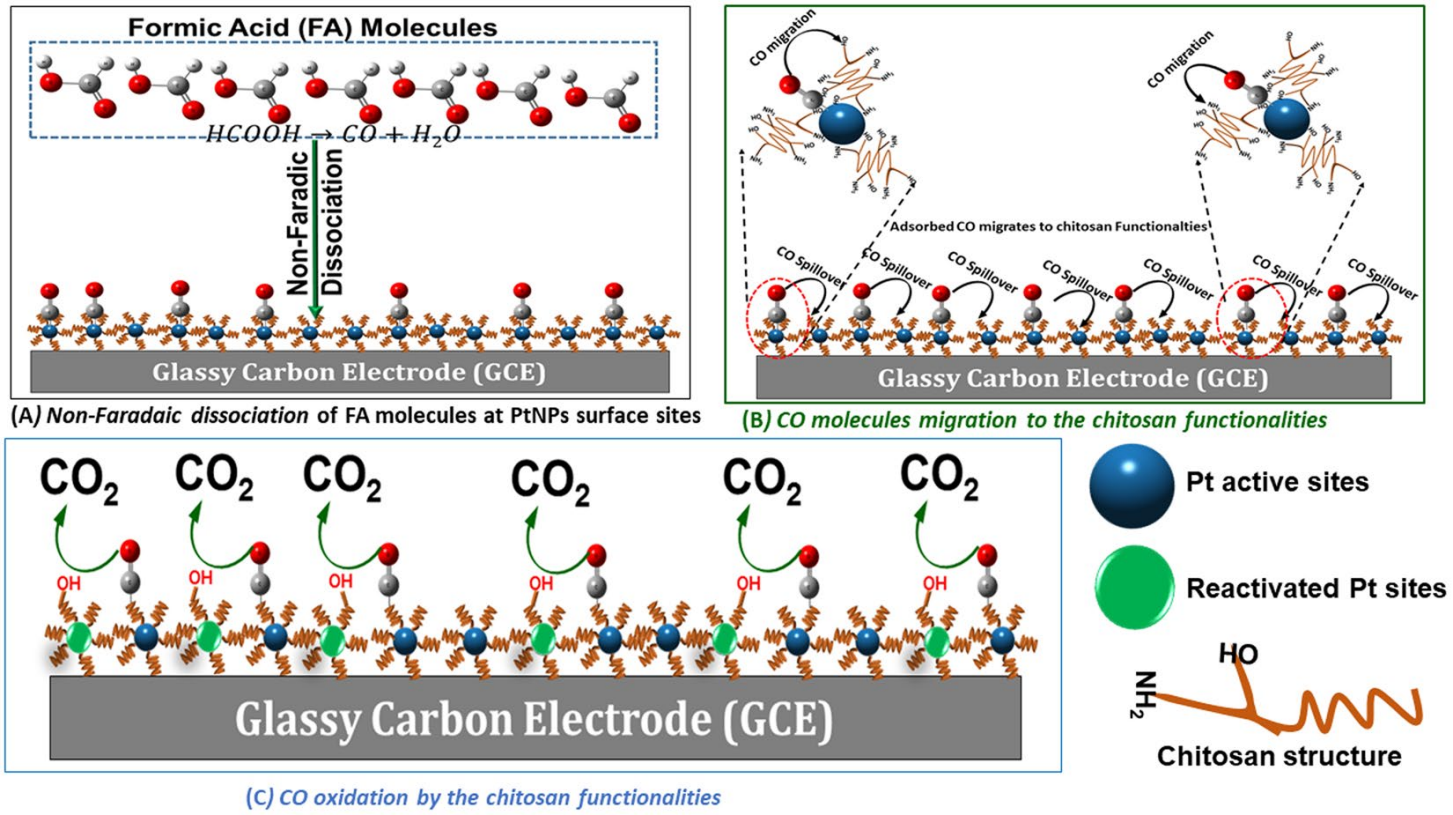
The proposed mechanism of FAO at nano-chitosan-PtNPs catalyst: CO spillover mechanism.
- Then, the most of the adsorbed CO molecules are believed to migrate from the PtNPs surface-sites to the chitosan functionalities (i.e., NH_2_ and OH-like groups) in a so-called “spillover phenomenon” which facilitates their oxidation at low potentials by a so-called *bifunctional effect* (Fig. 4). Additionally, the PtNPs active sites are believed to electronically interact with the surrounding chitosan shell (Fig. 5). This process retrieves the Pt active sites for FA adsorption again and increases the CO adsorption capacity. This can explain the appearance of only one CO oxidation peak and the significant negative shift of its onset potential at nano-chitosan-PtNPs catalyst (10% PtNPs, Fig. 3A-curve c).

Hence, the CO migration from the PtNPs active sites to the chitosan functionalities might be a plausible explanation according to the following scenario:

- Formic acid (FA) is first adsorbed/dissociated only on PtNPs active-surface sites resulting in CO and H_2_O formation, as indicated from curves a & b of Figs 2B and 3A.
- Next, the *in-situ* generated CO intermediates from the dehydration of FA are partially adsorbed at the PtNPs active-surface sites disabling the majority of them for the direct FAO (Fig. 4). This explains why the intensity of *I*_p_^ind^ is much higher than *I*_p_^d^ of both PtNPs (Fig. 2B-curve b) and commercial Pt/C (Fig. 3B-curve c) catalysts.

**Figure 5:**
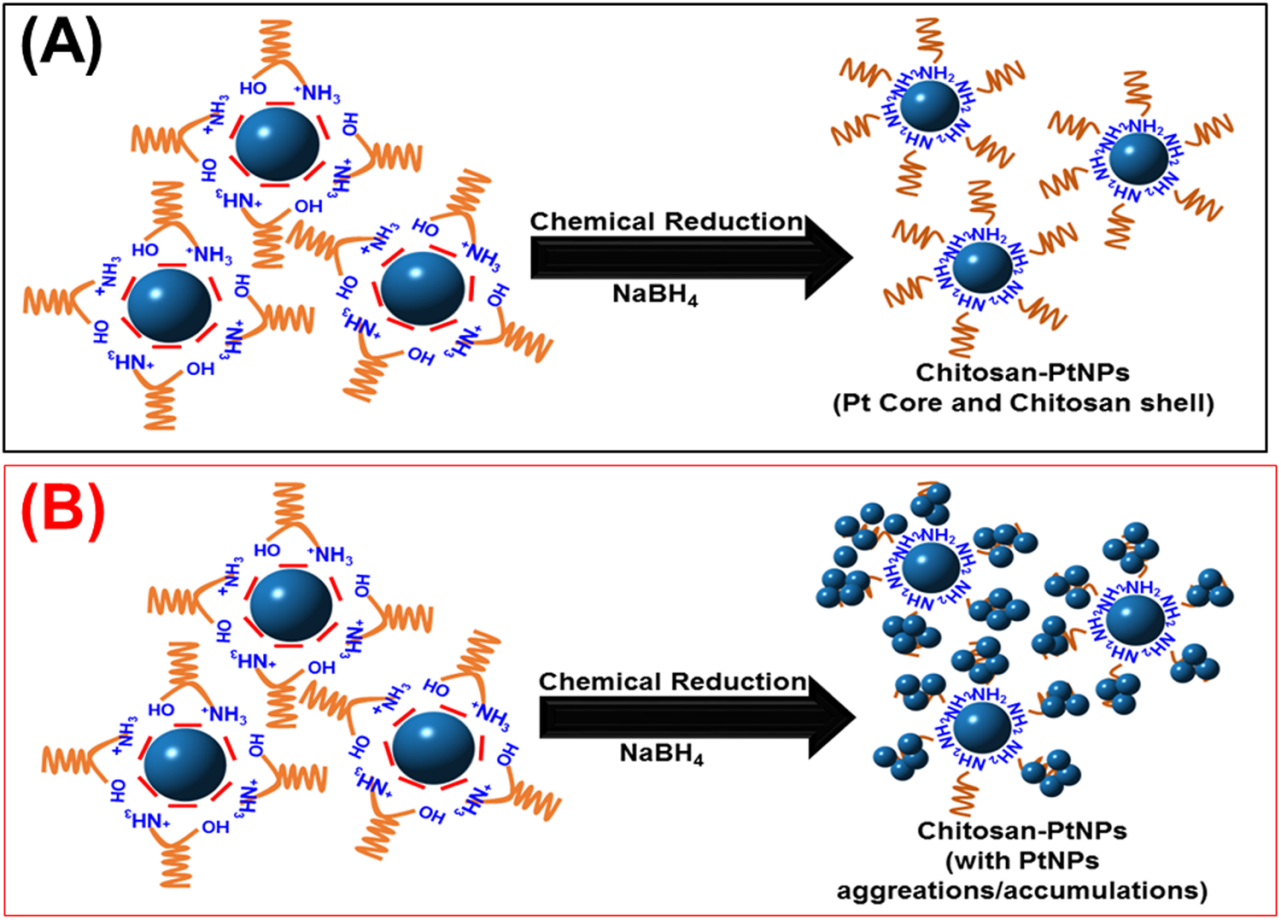
Schematic diagram shows the chitosan-PtNPs nanocomposites with (**A**) lower PtNPs weight % (less than 15%, exhibits core-shell structure with chitosan molecules shell) and (**B**) higher PtNPs weight percent (exhibits a core-shell structure with PtNPs aggregations atop the chitosan shell).
- Having explained the above, we need to clarify why two CO oxidation peaks will appear at the nano-chitosan-PtNPs catalysts with higher PtNPs weight percentages (18% ≤ Wt_Pt_% ≤ 30%, Fig. 3A-curves d,e). The amount of both embedded PtNPs and the generated CO molecules are believed to exceed the chitosan adsorption capacity. Thus, only some of the adsorbed CO molecules can migrate from the PtNPs to the nano-chitosan functionalities. This might explain the appearance of two different CO oxidation peaks; the first one (~0.28 V) is assigned to CO oxidation on the chitosan functionalities and the other one (~0.65 V) is attributed to CO oxidation at the PtNPs sites. N.b., the second oxidation peak is still negatively shifted relative to that of the pure PtNPs (in absence of chitosan; ~200 mV) indicating the electronic effect of the chitosan structure on the PtNPs surface sites.

That is, the amount of embedded PtNPs is believed to be much higher than the chitosan adsorption capacity, which results in the formation of PtNPs aggregates (as indicated from TEM image, see Fig. S5) atop of the chitosan matrix shielding the migration process of the adsorbed CO molecules to the chitosan sites (Fig. 5). N.b., the CO stripping peak is still negatively shifted compared to that of the pure PtNPs indicating the existence of the electronic interaction between the chitosan structure and PtNPs even at higher PtNPs loading. These observations are in good agreement with the formic acid oxidation CVs at the chitosan-PtNPs with higher PtNPs weight percentages (Fig. 3B), wherein the I_p_^ind^, referring to the oxidation of adsorbed CO, can only be observed when the wt_Pt_ % is greater than 15% and it significantly increases with the further increase of the wt_Pt_ %. On the other hand, the I_p_^d^ increases with the increase of the wt_pt_ % reaching its maximum at wt_Pt_ %~24%, beyond this value it decreases.

**Figure 6:**
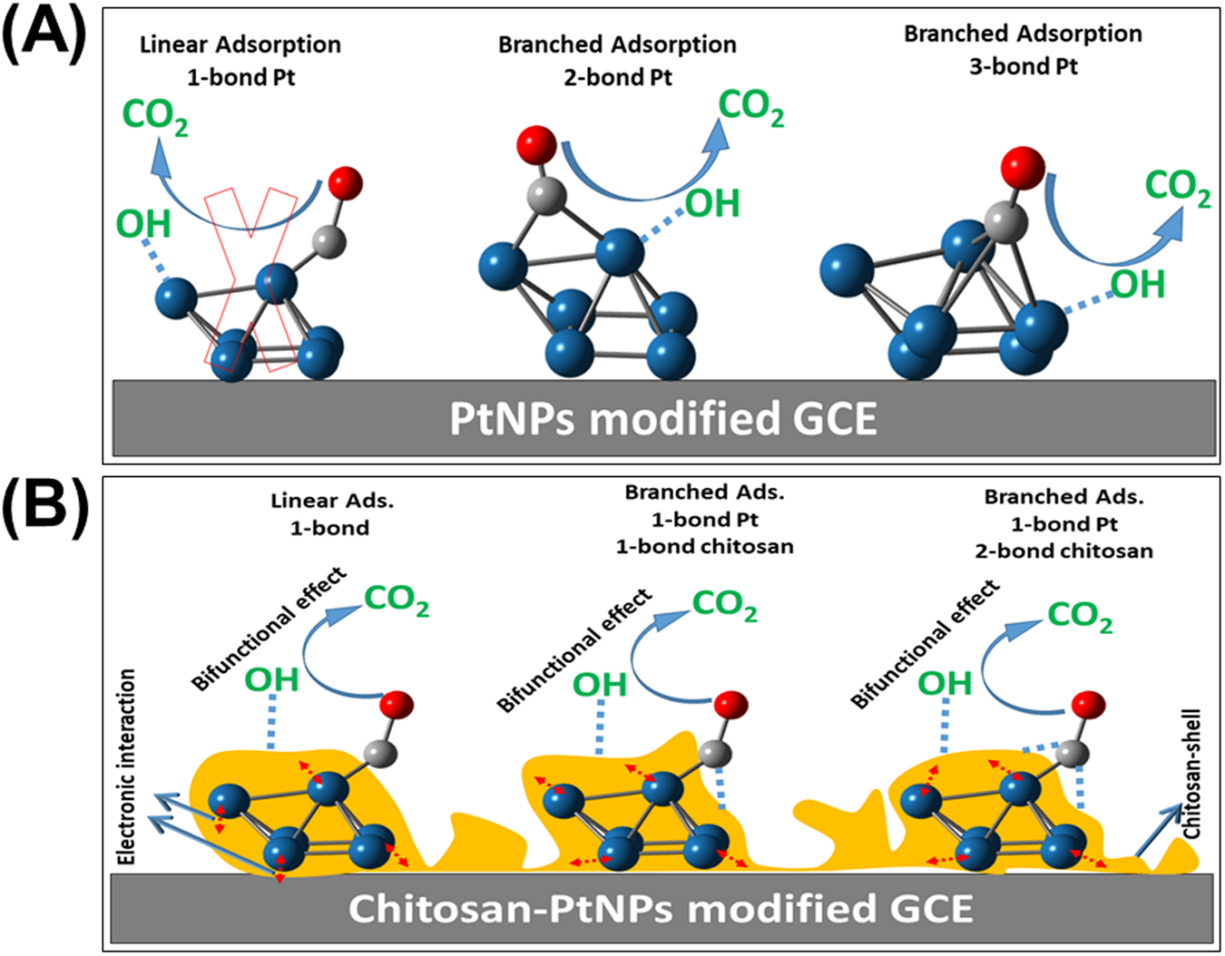
Schematic diagram shows the different adsorption modes of CO at (**A**) PtNPs and (**B**) nano-chitosan-PtNPs modified GCE.

The change of the CO adsorption mode from branched at pure PtNPs to linear mode on nano-chitosan-PtNPs could be another possible explanation for the increase of the amount of stripped CO from the nano-chitosan-PtNPs modified GCE surface compared to that of the pure PtNPs (in absence of chitosan). CO has two different adsorption modes atop of the Pt surfaces (namely; (a) linear adsorption mode (forms 1-bond with Pt) and (b) branched adsorption mode (forming 2 or 3 bonds with Pt), Fig. 6A). The number of adsorbed CO molecules per Pt surface-active site (CO:Pt) is calculated using the amount of charge associated with CO oxidative removal peak and hydrogen desorption peaks, as in our recently published article^1^, in order to determine the favorable CO adsorption in the absence and presence of the chitosan matrix. Each CO molecule requires 2.5 Pt sites in order to adsorb atop the pure PtNPs surface, suggesting the branched adsorption modes with 2 or 3 bonds with Pt are the favorable CO adsorption mode on the PtNPs surface (scheme 3A). On the other hand, each CO molecule requires only one Pt site to adsorb on the nano-chitosan-PtNPs suggesting the favorable linear adsorption mode (Fig. 6B) or assuming the branched CO adsorption mode as well, but with forming one bond with Pt and one or two bonds with chitosan functionalities (Fig. 6B).

### Stability Issue

**Figure 7:**
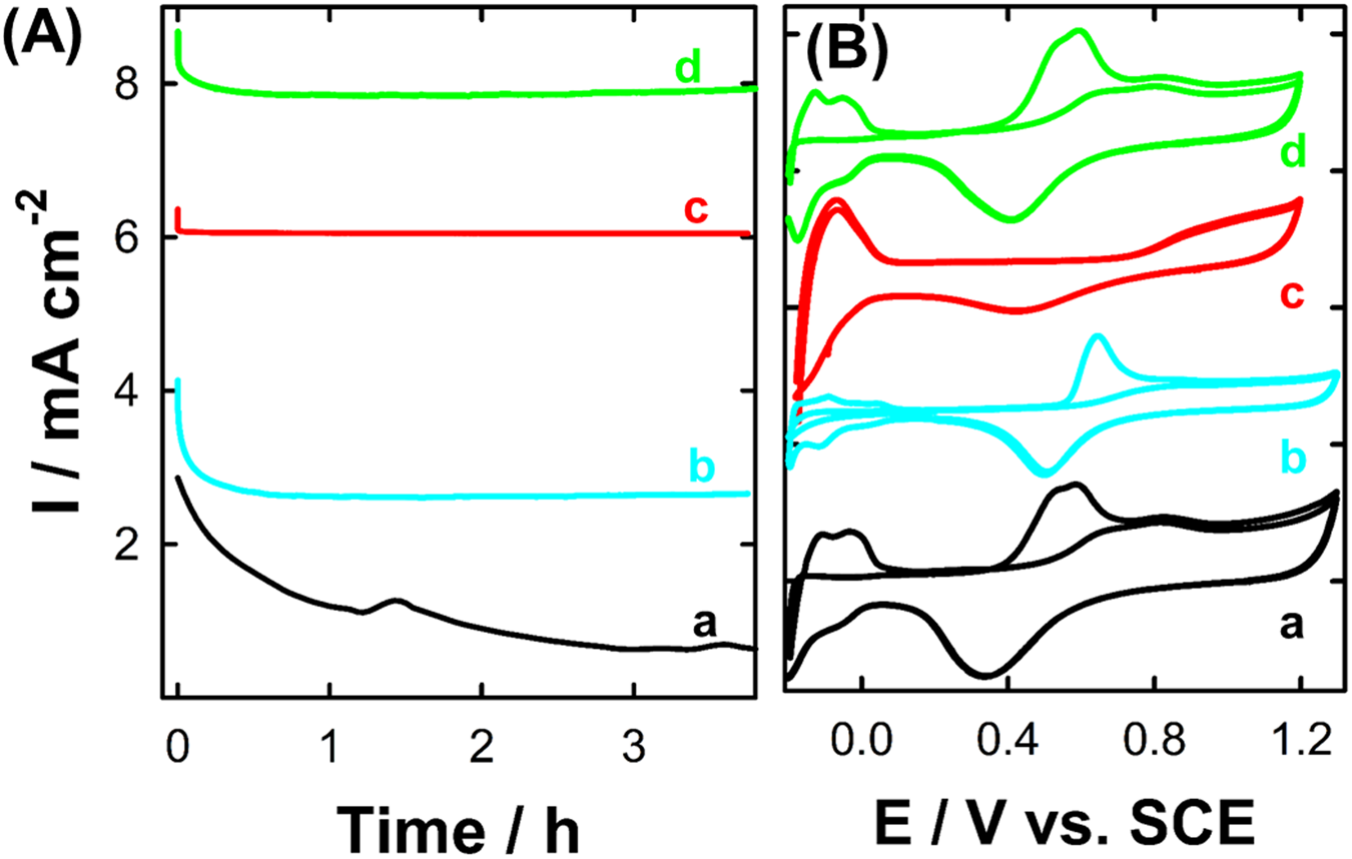
(**A**) chronoamperometric plots (i-t) obtained at the commercial Pt/C (curve a-black-line), PtNPs (curve b-cyan-line), nano-chitosan-PtNPs with 10% PtNPs (curve c-red-line) and nano-chitosan-PtNPs with 25% PtNPs (curve d-green-line) in 0.5 M H_2_SO_4_ containing 0.3 M FA. (**B**) CVs obtained at the same electrodes in 0.5 M H_2_SO_4_ after i-t measurements (same color code as in Fig. 5A).

Durability and stability of the catalysts are a central concern for any fuel cell manufacturer in addition to the catalyst’s activity. In a money saving concept, a stable electrode with a moderate efficiency (activity) is better than a highly active one with lower stability (fast performance degradation). That is, the moderately stable catalyst will live longer than the active catalyst with lower durability. Herein, the chemically embedded PtNPs inside the chitosan matrix are intended not only to improve the PtNPs catalytic activity, but also their long-term stability, which rapidly decays due to the significant accumulation of the CO poisoning intermediates at the PtNPs active surface sites together with their aggregation attributed to the corrosion of commercial carbon support^23,25^. The stability of the proposed electrodes was investigated by chronoamperometry for prolonged FA oxidation electrolysis time (up to 4 hours), data are presented in Fig. 7. The catalytic stability of both pure PtNPs (curve b) and the commercial Pt/C (curve a) modified GCE electrodes is rapidly diminished with time due to the deactivation of the Pt active surface sites by the adsorption and accumulation of the CO intermediates resulting from the *non-faradaic* dissociation of FA atop of Pt surface. That is, the PNPs and Pt/C modified GCE electrodes lost more than 65% and 40% of their original catalytic activities after only one hour of FA continuous electrolysis. On the other hand, this catalytic deterioration is effectively diminished at the chitosan-PtNPs/GCE (with PtNPs 15%, Fig. 7A-curve c), i.e., the measured catalytic stability remains effectively unchanged at a reasonably high value (only 4% catalytic stability loss), confirming the essential role of the chitosan matrix in improving the catalytic performance and CO poisoning tolerance of PtNPs for FA oxidation. Moreover, the chitosan-PtNPs/GCE with higher Pt weight percent (e.g., 23% Pt, see Fig. 7A-curve d) lost 12% of its initial catalytic activity, most likely due to the CO accumulation on the PtNPs surface.

In order to determine the origin of the improved stability of the chitosan-PtNPs catalyst, CVs were measured at the same above-used electrodes in Fig. 7 after carefully washing them with distilled water in 0.5 H_2_SO_4_, data are displayed in Fig. 7B. As seen in this figure, the CVs of both PtNPs and Pt/C modified GCE electrodes exhibited a broad oxidation peak around 0.6 V which is attributed to the accumulation of CO during the chronoamperometry measurement. The same behavior is observed for the chitosan-PtNPs with high Pt weight percentages (e.g., 23%). This explains the fast decay in their catalytic stability. Interestingly, there were no peaks observed for the chitosan-PtNPs/GCE (with 10% Pt), suggesting no CO is accumulated on their surface during the chronoamperometric measurement. This is the reason behind their high stability and activity compared to both pure PtNPs and the commercial Pt/C electrode. Additionally, the electrochemically active surface areas (ECSA) of both Pt/C and PtNPs modified GCE electrodes were significantly reduced after the chronoamperometry test (decreased by 40% and 53%, respectively), while ECSA of the nano-chitosan-PtNPs  remained effectively unchanged.

### DFT calculations

**Figure 8:**
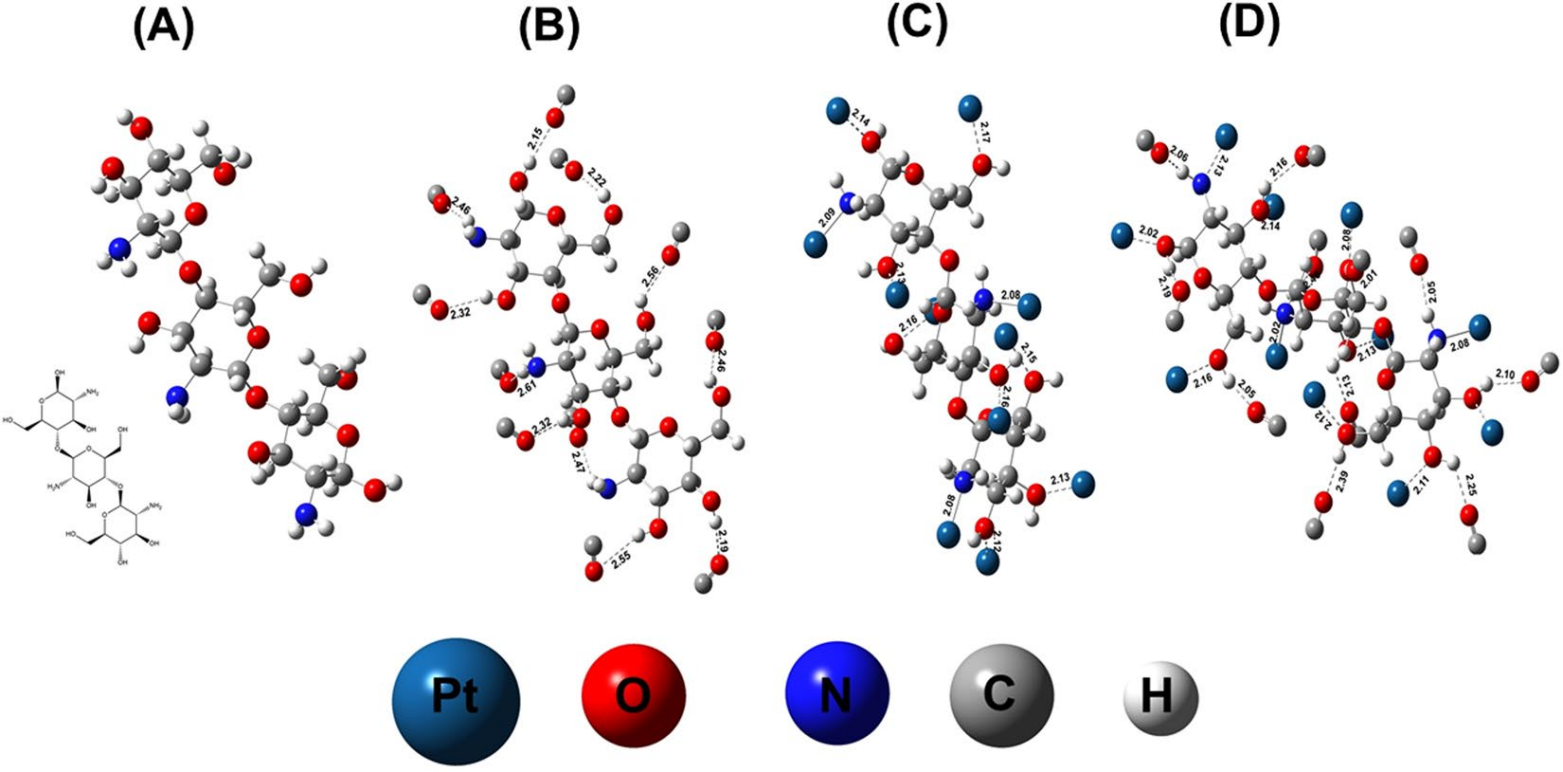
The optimized structures of (**A**) chitosan, (**B**) chitosan and 11 CO molecules, (**C**) chitosan and 11 Pt atoms and (**D**) chitosan with 11 CO molecules and 11 Pt atoms.

DFT calculations have been performed to model the interactions of PtNPs and/or carbon monoxide with the nano-chitosan matrix (see Fig. 8). Firstly, the chitosan structure is optimized in the absence of both PtNPs and CO (see Fig. 8A). Then, 11 CO molecules were added to the optimized chitosan structure in the absence of Pt atoms (see Fig. 8B) to examine the interaction between CO molecules and the chitosan functionalities. As clearly seen in this figure, the oxygen atoms of the CO molecules formed H-bond with the hydrogen atoms of the chitosan OH- and NH_2_- like functional groups, indicating the ability of chitosan functionalities to adsorb CO molecules. It is worth to mention that the CO-OH bonds are shorter compared to that of the CO-NH_2_ bonds. On the other hand, Fig. 8C shows the interaction between the Pt atoms with the chitosan functionalities, the Pt-N bonds are shorter than the Pt-O bonds. This supports our finding that chitosan forms a shell around the PtNPs stabilizing and preventing the PtNPs aggregations. Furthermore, Fig. 8D shows the addition of both Pt atoms and CO molecules at the same distances from the OH and NH_2_ functionalities of the optimized chitosan structure which is shown in Fig. 8A. As clearly seen in this figure, while the Pt atoms make bonds with the O- and N- of the OH and NH_2_ like functional groups, CO molecules prefer to form H-bonds with the hydrogen atoms of the OH- and NH_2_- functionalities. This might support our assumption of CO molecules spillover.

## Conclusion

This study introduces a highly efficient DFAFCs anode of a chitosan-PtNPs nanocomposite, wherein the PtNPs were embedded chemically inside the chitosan matrix. Chitosan-PtNPs nanocomposite catalysts exhibited higher electrocatalytic activity, catalytic stability together with a much better CO poisoning tolerance compared to that of the commercial Pt/C electrode. These outstanding enhancements are demonstrated by the exclusive oxidation of FA by the direct pathway (poisoning route completely diminished), significant negative shift of the FA oxidation onset potential (~400 mV) along with 23 times higher catalytic stability compared to the commercial Pt/C catalyst. Chitosan matrix (shell) is believed to protect the PtNPs from sintering and to increase their number of active surface sites. Besides, the chitosan functionalities are believed to retrieve the poisoned PtNPs sites with CO (produced from FA dehydration) via a so-called “*spillover mechanism*”, resulting in a significant increase in the catalysts CO-poisoning tolerance. Alternatively, the chitosan matrix is assumed to electronically interact with PtNPs in such a way that it weakens the CO-Pt binding and facilitates oxidative removal of the adsorbed CO at low potentials. This investigation presents a new way to improve the performance of the DFAFCs (e.g., activity and stability) even with using lower amounts of PtNPs (3 times lower) compared to the commercial Pt/C catalyst.

## Electronic supplementary material

Supplementary information
