# Supplementary material for: Efficient Direct Formic Acid Fuel Cells (DFAFCs) Anode Derived from Seafood waste: Migration Mechanism
Source: Sci Rep. 2017 Dec 19;7:17818. doi: 10.1038/s41598-017-17978-8 (PMC5736546; doi:10.1038/s41598-017-17978-8)
Supplement: Supplementary file 1 — Supplementary information [file 41598_2017_17978_MOESM1_ESM.pdf]

Efficient Direct Formic Acid Fuel Cells (DFAFCs) Anode Derived from Seafood waste: Migration Mechanism

Gumaa A. El-Nagar [a,b]* , *Mohamed A. Hassan* [c], *Iver Lauermann* [d] *and Christina Roth* [b]

[a] Chemistry Department, Faculty of Science, Cairo University, 12613 Cairo, Egypt
[b] Institute for Chemistry and Biochemistry, FU Berlin, Takustr. 3, D-14195 Berlin, Germany
[c] Nanotechnology and Advanced Materials Central Lab, Agriculture Research Center, Giza, Egypt
[d] Helmholtz-Zentrum Berlin für Materialien und Energie, Hahn-Meitner-Platz 1, 14109 Berlin, Germany

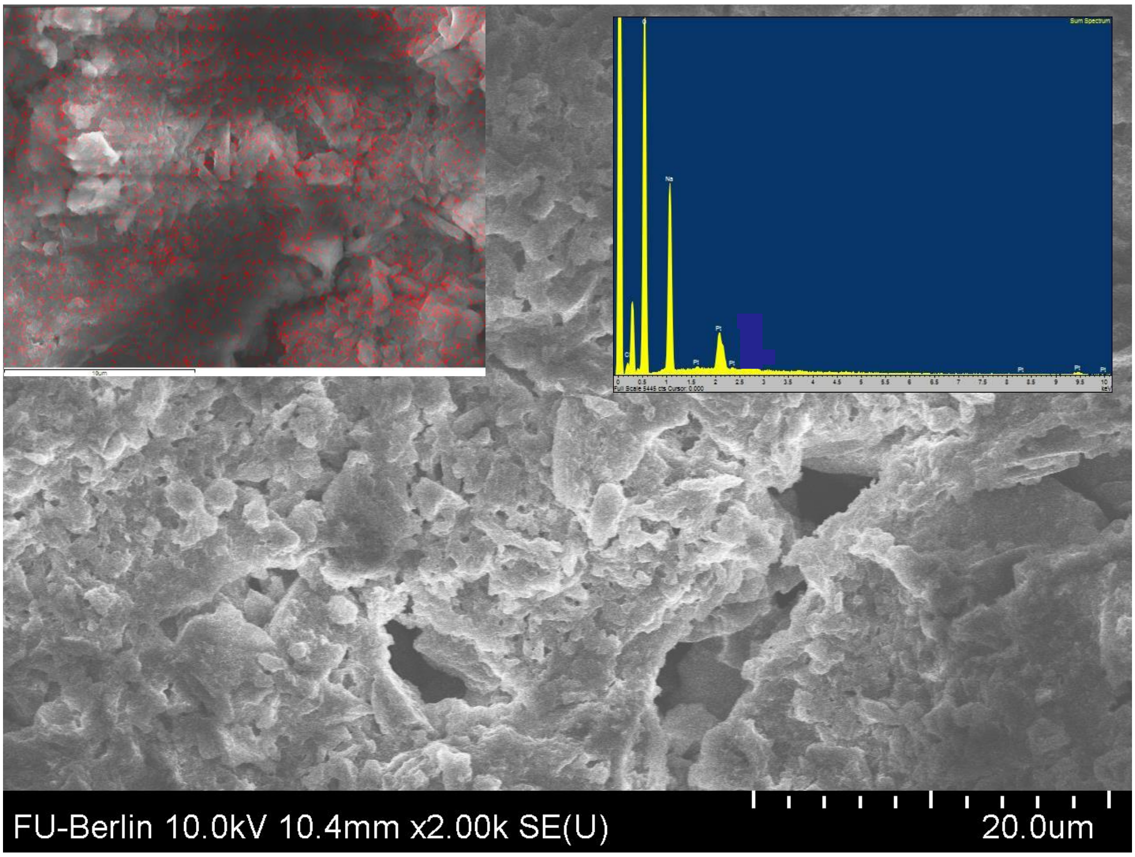

Fig. S1: SEM image of nano-chitosan-PtNPs. Insets are mapping EDS and EDS analysis of the same electrodes.

(A)

(B)

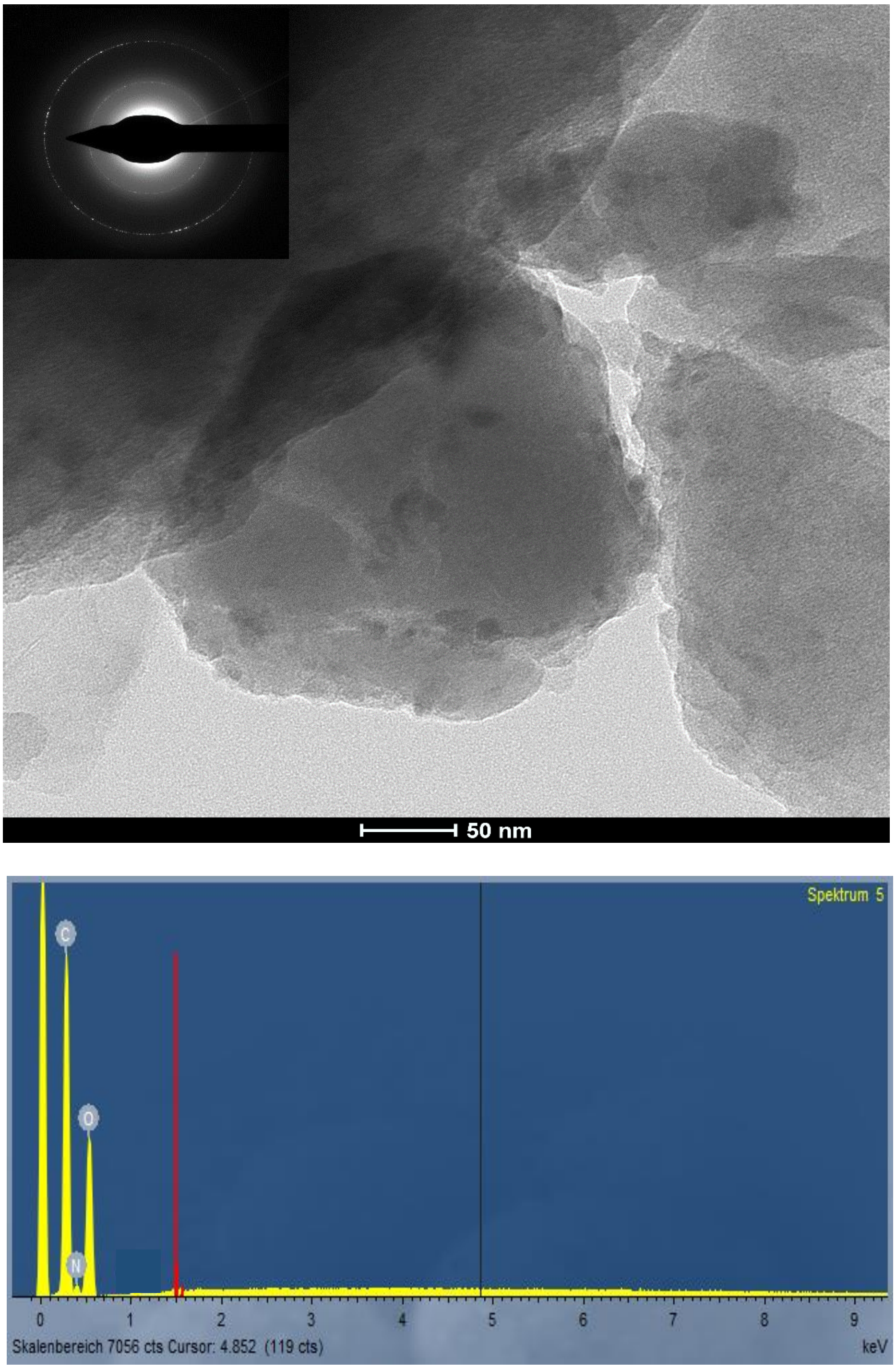

Fig. S2: (A) TEM image of nano-chitosan and (B) EDS analysis of nano-chitosan.

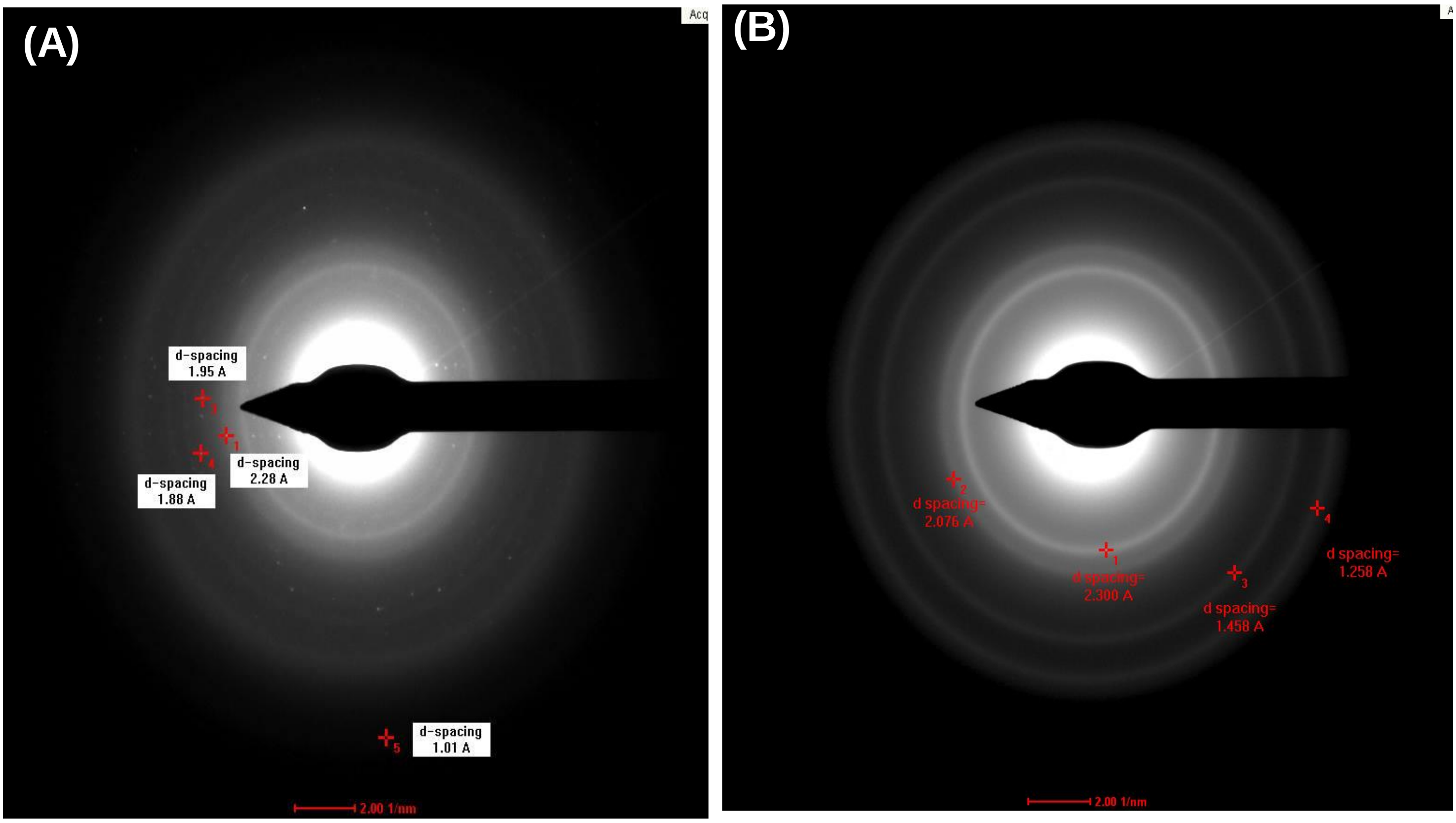

Figure S3: SAED patterns showing the lattice distances of both commercial Pt nanoparticles (A) and Chitosan-PtNPs nanocomposite (B).

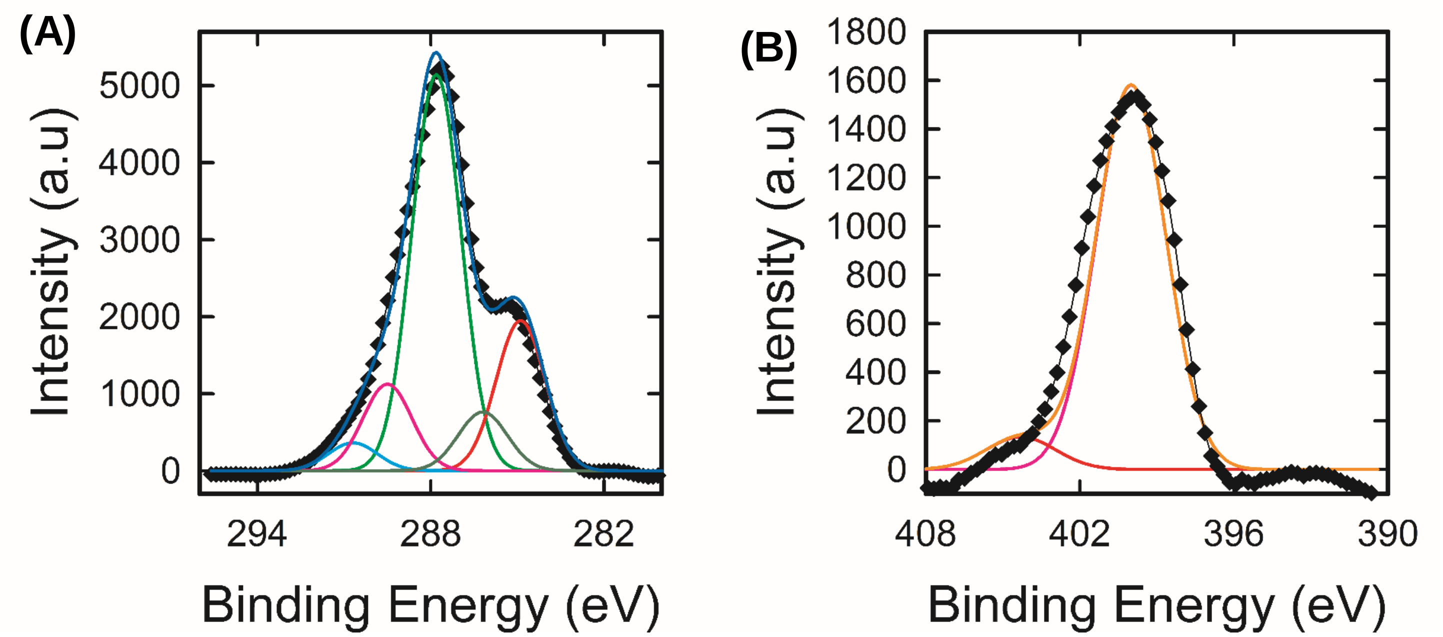

Figure S4: High resolution XPS spectra of (A) C1s and (B) N1s of chitosan-PtNPs nanocomposite.

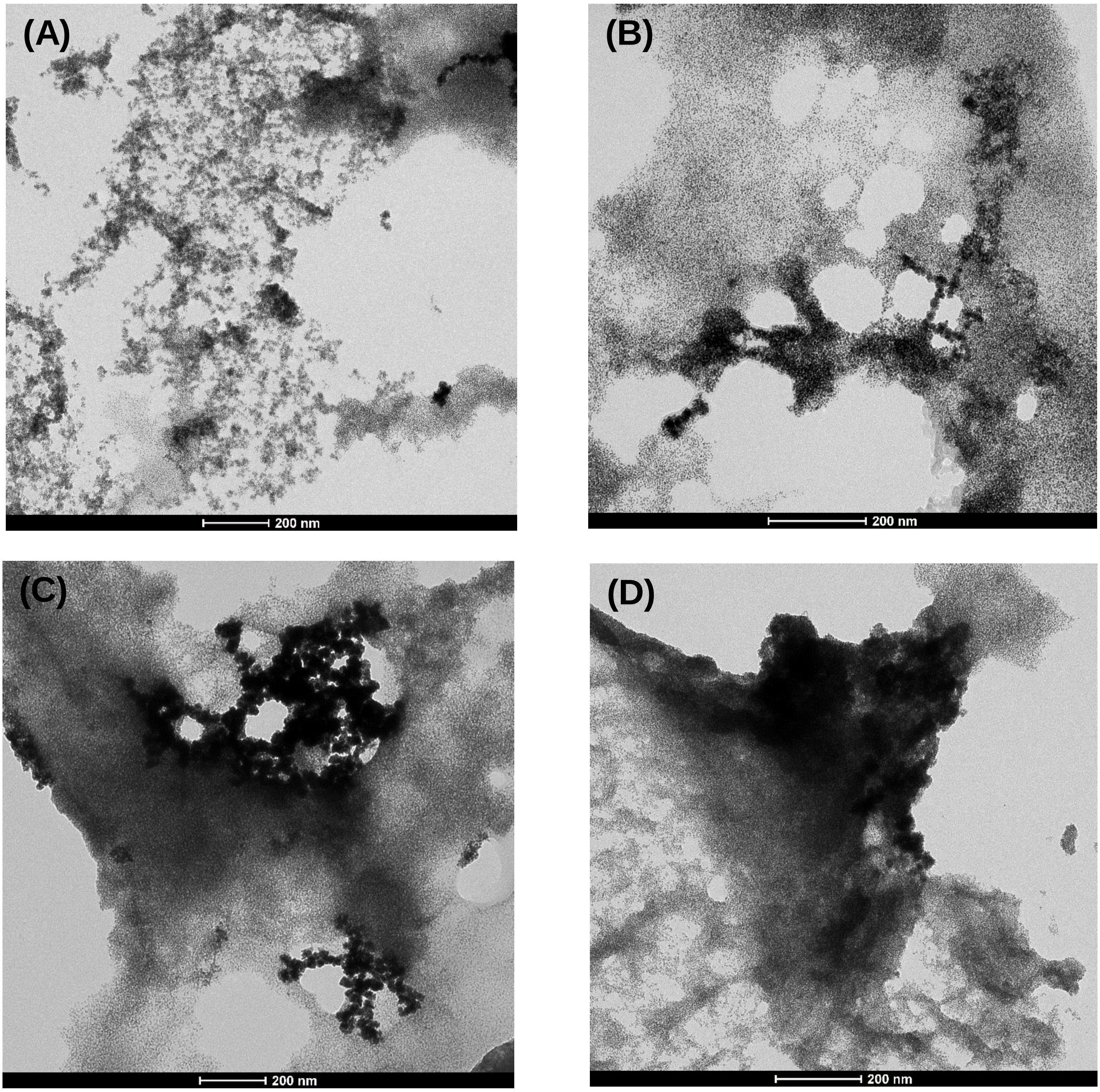

Fig. S5: TEM images of nano-chitosan with higher PtNPs weight percent ($Wt_{PtNPs}\geq15\%$) showing the aggregations/accumulations of PtNPs atop of the chitosan matrix shell.

Table S1: A comparison of the electrocatalytic activities reported for different catalysts towards formic acid oxidation

Electrode	I_p^d/I_p^{ind}	I_p^d/I_b	Onset potential (mV vs SCE)	Stability loss	References
Commercial PtC	0.35	0.20	+500	40 %	This work
PtNPs	0.31	0.13	+560	65%	This work
Chitosan-PtNPs	840	1.0	-250	3%	This work
Pt/PANi/GC	15.5	1.0	+190	13%	[1]
Pt-Cu/C	-	2.3	+156	34%	[2]
Pt-Cu-Fe/C	-	12.4	+200	50%	[2]
Pt-Li	1.0	0.50	+150	-	[3]
Pt-Ru/PCZ/GC	-	1.4	-100	16%	[4]
Pt–Ru/GC	-	0.90	+100	60%	[4]
AgPt octahedra	2.77	-	+200	50%	[5]
nano-Cu_2O–Pt/GC	-	0.93	-100	4%	[6]
D-PtTe-3-700	-	0.99	+70	50%	[7]
Pt1Ru3/NG	-	0.99	+50	67%	[8]

Note that, the high I_p^d/I_p^{ind} ratio indicates the high electrocatalytic activity of the catalyst for direct formic acid oxidation to CO2. Additionally, as the Ipd/Ib ratio close to 1.0 indicates the high CO tolerance of the catalyst material. Some of the above numbers are calculated based on the results reported in their respective articles.

1. Abd El-Moghny, M. G. et al. Conducting polymers inducing catalysis: Enhanced formic acid electro-oxidation at a Pt/polyaniline nanocatalyst. *International Journal of Hydrogen Energy* **42**, 11166-11176 (2017).
2. Wang, Y.-X. et al. Concave Pt-Cu-Fe ternary nanocubes: One-pot synthesis and their electrocatalytic activity of methanol and formic acid oxidation. *Chinese Chemical Letters* **28**, 60-64 (2017).
3. Awaludin, Z., Okajima, T. & Ohsaka, T. Formation of Pt–Li alloy and its activity towards formic acid oxidation. *Electrochemistry Communications* **31**, 100-103 (2013).

4. Zhou, W., Xu, J., Du, Y. & Yang, P. Polycarbazole as an efficient promoter for electrocatalytic oxidation of formic acid on Pt and Pt–Ru nanoparticles. *International Journal of Hydrogen Energy* **36**, 1903-1912 (2011).
5. Jiang, X. et al. Porous AgPt@Pt Nanooctahedra as an Efficient Catalyst toward Formic Acid Oxidation with Predominant Dehydrogenation Pathway. *ACS Applied Materials & Interfaces* **8**, 31076-31082 (2016).
6. El-Nagar, G. A., Mohammad, A. M., El-Deab, M. S. & El-Anadouli, B. E. Propitious Dendritic Cu2O–Pt Nanostructured Anodes for Direct Formic Acid Fuel Cells. *ACS Applied Materials & Interfaces* **9**, 19766-19772 (2017).
7. Zhou, F. et al. The study of platinum-tellurium intermetallic nanoparticles for formic acid electro-oxidation. *Electrochimica Acta* **248**, 307-312 (2017).
8. Xu, H. et al. One-pot fabrication of N-doped graphene supported dandelion-like PtRu nanocrystals as efficient and robust electrocatalysts towards formic acid oxidation. *Journal of Colloid and Interface Science*.
